# PROTOCOL: Social and behaviour change communication interventions for strengthening HIV prevention and research among adolescent girls and young women in low‐ and middle‐income countries: An evidence and gap map

**DOI:** 10.1002/cl2.1211

**Published:** 2022-01-18

**Authors:** Devi Leena Bose, Anhad Hundal, Kuhika Seth, Saif ul Hadi, Sabina Singh, Shweta Singh, Ashrita Saran, Kriti Goyal, Jessy Joseph, Solomon Salve

**Affiliations:** ^1^ IAVI New Delhi India; ^2^ Campbell Collaboration South Asia New Delhi India

## Abstract

Despite progress in several dimensions of the global HIV response, there seems to be a significant gender and age disparity. Numerous organizations consider it a top priority to accelerate HIV prevention programming among Adolescent Girls and Young Women (AGYW) as unequal gender norms, limited agency and voice, and reduced access to resources put them at higher HIV risk. Gender and age have also been identified as critical gaps within prevention research to ensure the development of biomedical interventions that are responsive to the biological and social needs of AGYW. Towards this, the objectives of the proposed evidence and gap map are to; identify and map existing evidence and gaps on the use of diverse Social and Behaviour Change Communication (SBCC) strategies to strengthen adoption of HIV prevention measures and participation in research among AGYW in LMICs; and, identify areas where more interventions and evidence are needed to inform the design of future SBCC strategies and programs for AGYW engagement in HIV prevention and research.

## 1 BACKGROUND

### 1.1 Introduction to problem

Choices made during adolescence and as young adults continue to shape behaviours and health choices well into adulthood. Methodologies centred on behavioural change have proven to be effective in the adoption and adherence of better health seeking practices. While the global buy‐in for socio‐behavioural interventions to support demand and uptake of HIV services is on the rise, evidence mapping of the scope and impact of this in the context of Adolescent Girls and Young Women (AGYW) needs to be systematically outlined and enumerated to help further adoption by researchers, policy makers and HIV program managers.

Converging social, cultural and economic factors affect the way in which adolescent girls and young women understand, negotiate and access information and biomedical treatment related to HIV. Persistent gender and age disparities, and stigmas around female sexuality faced by this group in the impact of HIV are both exacerbated by, and reinforce issues such as poverty, lack of access to education (including sexual and reproductive health education) and livelihood opportunities, limited financial autonomy, lack of access to sexual and reproductive healthcare (as well as other healthcare services), and the risk of violence, including intimate partner violence. The National Family Health Survey of India (NFHS‐4), 2015–2016 (2017), for instance, highlights gender‐based gaps in awareness levels and health seeking behaviours; while 75% of young women surveyed had heard of HIV, only 22% had comprehensive knowledge about the disease, a marginal increase from the previous 20%; While approximately 44% of young women knew where to get tested, it differed depending on indicators such as age, wealth, education, residence, marital status, caste and religion. Furthermore, the adoption of family planning methods was lower among adolescent girls than young women between the ages of 20–24 years, highlighting the requirement of more spotlighted and intensive education for that age group.

Because of these converging factors, many organizations have put AGYW at the centre of their response to HIV, including UNAIDS (2016), UNFPA (2016), World Health Organization (2017) and national bodies like the National AIDS Control Organization (NACO) (n.d.). According to UNAIDS (2019), 6200 women and adolescent girls between the ages of 15–24 years are newly infected by HIV every week, and 50 adolescent girls die from HIV related diseases every day, including treatable diseases like cervical cancer (Stegling, 2019).

There is a greater need for active engagement in prevention research to ensure the development of biomedical interventions that are responsive to both the biological and social needs of AGYW.

Here, then, Social and Behaviour Change Communication (SBCC) interventions form a pivotal part of the comprehensive HIV prevention response, as they not only help address aspects of underlying behavioural and social barriers that drive the epidemic, but also play a critical role in expanding knowledge of, and access to, quality health services, strengthen equity and participation and assist in addressing gendered health disparities throughout the continuum of care (Centres for Disease Control and Prevention [CDC], 2017), thereby creating an enabling environment. However, there is also an understanding that, over the years, health communication has remained underutilized; not only is health communication often included into programs as an afterthought rather than integrated right in the beginning, the evidence towards creating this enabling environment, whereby uptake and support of prevention options and research is key, is often scattered (Sugg, 2016).

### 1.2 The intervention

An evidence and gap map (EGM) is a decision making and research prioritization tool that highlights gaps in research to inform strategic health and social policy, program and research priorities (Snilstveit et al., 2013). Simply put, it provides a visual display of what we know and do not know by highlighting evidence that is either strong, weak or non‐existent.

EGMs are useful tools in avoiding needless duplication, and can also be used to identify where sufficient, high‐quality evidence from systematic reviews and randomized trials are available as a basis for decisions or where sufficient studies are available for knowledge synthesis (Snilstveit et al., 2016).

This EGM is important to consolidate existing evidence about SBCC strategies for AGYW in Low and Middle‐Income Countries (LMICs), as well as understand the various behaviours they target, thereby identifying areas where more evidence and interventions are needed.

This will help our work by situating the role of Experiential Learning and inform the design of future strategies. It is aligned with the Intervention‐Outcome Framework, whereby; (1) Intervention would be limited to SBCC interventions defined as the 'strategic use of communication approaches to promote changes in knowledge, attitude, norms, beliefs and behaviours' (Johns Hopkins University, 2016), and, (2) Outcomes would be categorized under two broader levels—(a) dimensions of behaviour change, and, (b) social and health outcomes. Behaviour change dimensions would be guided by the COM‐B model (Capability, Opportunity, Motivation and Behaviour model) and the Theoretical Domains Framework (TDF) and would be broadly categorized under Capability, Motivation and Opportunity. The health and social outcomes will be defined based on programmatic and research priorities as defined by UNAIDS, Global Fund and USAID.

We have a broad range of intended user groups including scientists, community engagement practitioners, socio‐behavioural researchers, policy and decision makers, intervention designers and the general public. We will also be engaging with stakeholders who will play a critical role in helping define the scope, conceptual framework and methodology. Further, they would be consulted to assist in reviewing the preliminary results, drafting the report and further promoting the use of the map both at implementation and policy level.

## 2 OBJECTIVES

The study would undertake a comprehensive mapping of the evidence on the use of various SBCC intervention strategies globally targeted at AGYW (15‐24 years) and assess the gaps in evaluation of these strategies for HIV prevention and research.

Through this endeavour we aim to:

- Identify and map existing evidence and gaps on the use of diverse SBCC strategies to strengthen adoption of HIV prevention measures and facilitate participation in research among AGYW in LMICs.
- Identify areas where more interventions and evidence are needed to better inform design of future SBCC strategies and programs for AGYW engagement in HIV prevention and research.

## 3 SCOPE

### 3.1 Conceptual framework for SBCC interventions for strengthening HIV prevention and research

SBCC uses behaviour centred communication approaches to help inform, persuade and provide support to individuals, households, peers and communities towards creating an enabling environment across continuum of HIV care beginning with prevention (Van Lith & Mallalieu, n.d). SBCC strategies are informed by multiple disciplines including social sciences, behavioural sciences, behavioural economics, human centred design and other theories of behaviour change that address various determinants of behaviour and the environment within which change occurs. SBCC interventions can be further categorized under three broad strategies: social and community mobilization, behaviour change communication and advocacy (Lamstein et al., 2014).

**Figure 1:**
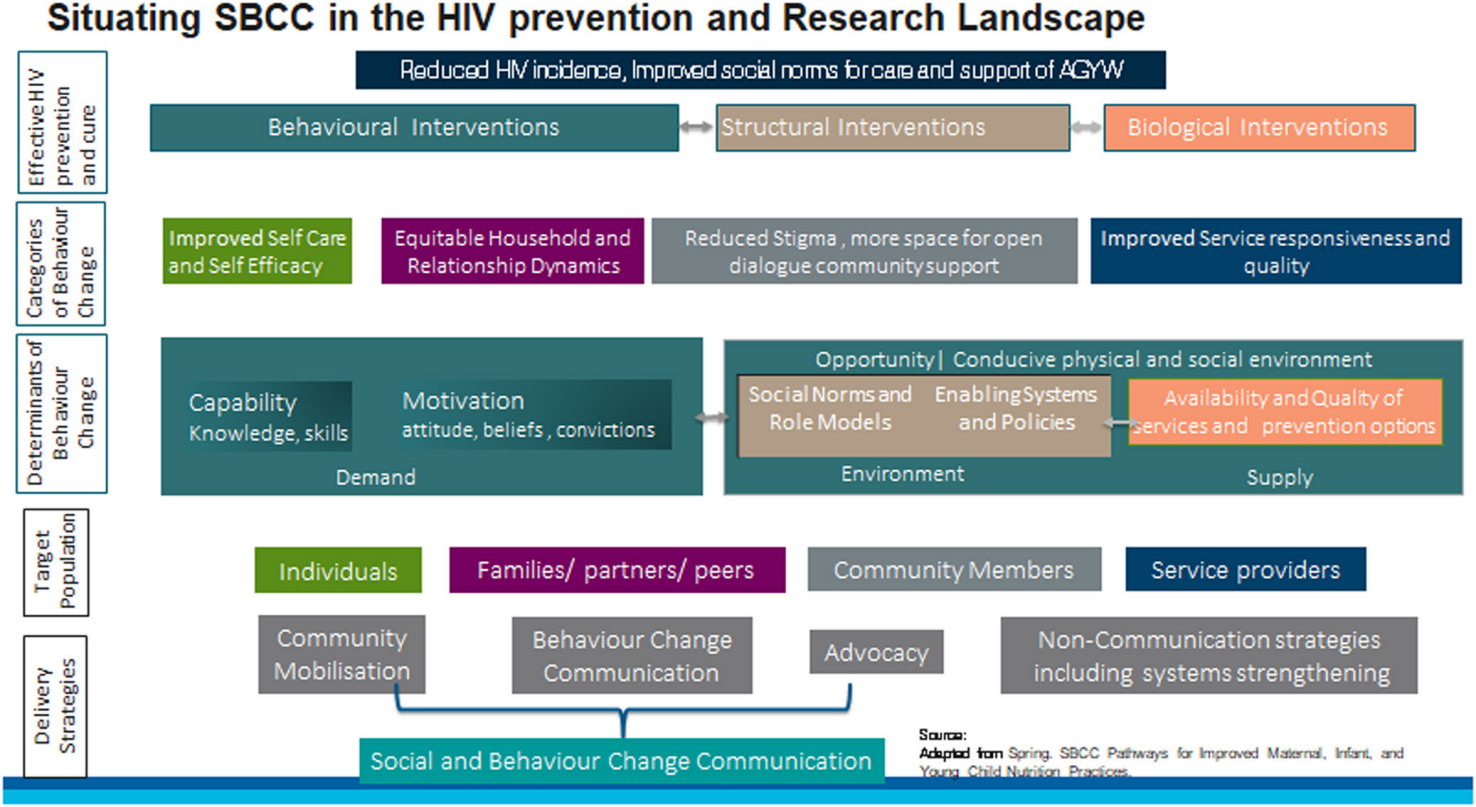
Conceptual framework

The conceptual framework below (Figure 1) illustrates how SBCC strategies can contribute towards navigating behavioural pathways within complex systems to ensure global objectives of reduced HIV incidence and improved social norms for care is achieved for AGYW population in LMICs.

Towards achieving reduced HIV incidence, improved social norms and support to AGYW we need a mix of behavioural, structural and biological interventions. The success of these interventions is dependent on multiple intrinsic and extrinsic behavioural factors constantly interact with each other. Towards delineating some of these factors we used the COM‐B model (Capability, Opportunity, Motivation and Behaviour model) proposed by Michie et al. (2014) and the Theoretical Domains Framework (TDF), which offers a comprehensive framework to identify factors that may directly influence behaviours, as well as desired behaviour changes (Khan & Roche, 2018). This was further categorized based on the work of Marsh et al. (2008) who suggested that adoption of a behaviour is dependent on three basic determinants—demand, environment and supply. Within all this, it is also important to mention the range of uncertainties and risks related to the development of a society in general, relating to technological advancements, urbanization, and so forth (Beck, 2013). All these dynamisms brought in by modern society, as well as contingencies, have a bearing on behaviours, the most recent being COVID‐19 pandemic. While these lie beyond the scope of the current EGM, some of this has been touched upon in the EGM report.

Given the complex systems within which behaviour change happens, we identified four key target groups that can have direct or indirect influence on individual's agency and decision‐making power for adopting/willingness for change. These include AGYW individuals and those with who they are in immediate relation, for example, family, partner, peers, and so forth, and actors who might be outside immediate circle of influence and yet can significantly influence decision making, for example, community members like religious and key opinion leaders, service providers, and so forth.

Lastly the framework focuses on SBCC delivery strategies, which are a mix of community mobilization, behaviour change communication and advocacy strategies. While other noncommunication strategies, for example, delivery and access to services are also critical for behaviour change, the focus of this EGM remains on SBCC‐based delivery strategies. Further, it is difficult to delineate how much of health communication translates into SBCC, especially those relating to structural factors, but there are significant intersections and overlaps that this EGM will focus on.

### 3.2 Overview of the scope of the EGM on SBCC interventions for strengthening HIV prevention and research among AGYW in LMICs

The proposed EGM focuses on mapping evidence available on the effectiveness of various kinds of SBCC interventions used for engaging AGYW in HIV prevention and research and help understand behavioural determinants targeted by these interventions among AGYW and their influencers leading towards desired social and health outcomes.

Based on the above‐mentioned framework, the substantive scope of the EGM is delineated along the following two key categories:

1. Interventions consisting of SBCC intervention strategies.
2. Outcomes consisting of the behavioural outcomes, health outcomes and research outcomes. Behaviour change dimensions as mentioned above were guided by the COM‐B model and The Theoretical Domains Framework (TDF). Health and research outcomes have been defined based on programmatic and research priorities as defined by UNAIDS (2020a, 2020b), The Global Fund (2018), and the World Health Organization (2020).

To keep the scope manageable the EGM would focus on the following topics of interest around HIV/AIDS and other STIs:

- Testing and uptake of prevention options including condoms, PrEP (pre‐exposure prophylaxis), PEP (postexposure prophylaxis), and long acting antiretroviral (ARV) drugs and other available or upcoming prevention options.
- Consistent uptake of Antiretroviral Therapy (ART).
- Participation in biomedical research.
- Intimate partner violence, sexual violence or gender‐based violence affecting HIV/SRH (sexual and reproductive health) related health outcomes.
- Behavioural factors affecting access to sexual and reproductive health services, such as relationship goals, sexual negotiations, gaps in knowledge, self‐efficacy, risk perception.
- Stigma and discrimination related barriers.
- Collective action, community support, community‐driven action and social and gender norms affecting SRH and HIV related behaviours.

The EGM will *not* focus on other structural factors affecting HIV acquisition in AGYW population like school attendance, labour migration, child sexual abuse, lack of economic empowerment, structural barriers to accessing sexual and reproductive health services, marriage patterns, and so forth. In addition to these biological factors, other adolescent health issues like mental health, smoking, alcoholism, drug addiction, nutrition, and so forth, maternal health and childcare would be out of scope. Also, topics related to pregnancy, family planning, abortions would be out of scope if they do not measure effects of HIV/SRH outcomes. It is important to acknowledge that these are also extremely critical and encourage those working on the above domains to investigate these to have a more holistic picture.

The details of interventions and outcomes included in the study have been outlined in detail in the next section.

#### 3.2.1 Population

The study focuses on ‘Adolescent Girls and Young Women' as defined by UNAIDS, between the ages of 15–24 in ‘low and middle‐income countries', and their influencers. LMICs are defined by World Bank as low‐income economies—those with a Gross National Income (GNI) of $1035 or less in 2019; lower middle‐income economies—those with a GNI per capita between $1036 and $4045; and upper middle‐income economies—those with a GNI per capita between $4046 and $12,535 (2020).

##### Sub‐population

Urban and rural AGYW in formal and informal settlements (according to United Nations Millennium Development Goals Report (2012) formal settings are characterized by adequate spatial planning and utilities (water, electricity, telephone, road and other infrastructure) and informal areas with no urban development (e.g., slums); in the rural context, informal settings can be tribal areas; informal settings can also include refugee camps in conflict or disaster affected areas). For the purpose of this study we will be looking at four kind of settings—urban formal (formal housing settlements, formal institutions like school/college, hospital, religious places, office and other areas under the municipality, etc.); urban informal (slums/unauthorized settlements), rural formal (formal settlements and institutions like local governance system, health centres, hospitals, religious institutions, commercial farms, community‐based organization [CBOs], schools, etc.) and rural informal (including community spaces like market areas, public meeting grounds, tea stalls, community fair grounds, etc.). Additionally, informal settings can also include refugee camps in conflict or disaster affected areas in both urban and rural context. In addition to these, the EGM would focus on women living in a high‐risk environment, for example, sex workers, intravenous drug users, and so forth.

Further, the EGM would also include the influence networks of AGYW, primarily male partners, family elders, peers, key community members and service providers.

### 3.3 Intervention and outcome framework

The Intervention and Outcome framework was drafted based on existing literature as mentioned in the above section. Further, in consultation with advisory group intervention and outcome categories were agreed and used to define the final scope of the EGM. The Group of Advisors (GoA) comprise of nationally and internationally recognized domain experts, including bioethicists, researchers, scientists, social scientists, experts in health education, gender mainstreaming, Good Participatory Practices (GPP), as well as advocacy and communication professionals. Experts will be consulted at four key stages:

1. At the inception to define the scope and review the draft conceptual framework.
2. Review of search methods and strategy.
3. Review preliminary results.
4. Review draft report.

A detailed list of the advisors and experts helping in different stages has been added in the Supporting Information Appendix A.

#### 3.3.1 Intervention categories

Based on the above‐mentioned scope (Section 3.2) for categorizing interventions the focus was on SBCC based interventions. A thorough review of academic and policy literature (Behaviour Change Impact, n.d.; Health Communication Capacity Collaborative, n.d.; SPRING, 2014; Tomori et al., 2014) was undertaken to curate a list of relevant SBCC interventions. The intervention list was further finalized based on inputs from the group of experts.

The SBCC interventions focussed on four key categories—mass media‐based, community media‐based, interpersonal communication and digital media based. These interventions were further categorized based on various SBCC interventions referenced in: the Bill and Melinda Gates Foundations 'Social & Behaviour Change Interventions Landscaping Study: A Global Review' (Storey et al., 2011), SBC for HIV Evidence database (Behaviour Change Impact, n.d.), C‐Change/FHI360 Learning Package for Social and Behaviour Change Communication (C‐Change, 2012), Health Communication Strategies in Combination HIV Prevention and Care Programs (Vermund et al., 2014), and Evidence of Effective Approaches to Social and Behaviour Change Communication for Preventing and Reducing Stunting and Anaemia (SPRING, 2014) (Table 1).

**Table 1:** Intervention categories

Intervention	Intervention definition
*Mass media interventions*	
*Interventions which are characterized by the expansive reach and are intended to reach a mass audience fall under mass‐media. These are large‐scale and usually quite cost‐intensive*.	
Print media	Interventions that use print‐based material, such as books, newspapers, journals, comics, novels, posters and brochures to disseminate textual and visual information to diverse audiences on a large scale.
Electronic media	Interventions that use audio‐visual material, such as TV and radio to disseminate textual and visual information to diverse audiences on a large scale.
*Community‐based interventions*	
*Interventions which are designed for/with the community where the key population of interest resides. In this, interventions are more localized and contextualized and aim at achieving a community buy‐in*.	
Community media	Community media‐driven interventions—such as community radio, video, newspapers, newsletters, and community screenings—that encourage participation of individuals, groups or organizations through locally established and geographically specific media forms.
Folk media	Interventions that use localized, traditional media in the form of music, drama, dance and puppetry.
Theatre and arts‐based approaches	Interventions that use contextualized dramatic art forms to prompt community participation on specific issues.
Community dialogues	Interventions that initiate open discussions and dialogues among participants and local groups through facilitated sessions that support self‐reflection around issues pertaining to HIV/SRH.
Capacity strengthening	Interventions targeted at strengthening knowledge, skills and capabilities of both providers and recipients of health services to adopt/deliver health interventions effectively.
Gamification and experiential learning	Interventions that support people‐centric, hands‐on learning, through pedagogies such as game components, participatory learning including the use of score, challenge, and achievement to motivate and engage participants (Kolb, 1984).
*Interpersonal communication*	
*Interventions that involve one‐to‐one or small group interaction and exchange*.	
Counselling	Interventions that use one‐on‐one communication with a trusted communicator or community leader such as a counsellor, teacher or health provider.
Home visits	Interventions where the homes of target populations and their influencers are visited by peers and community health workers to engage with them in a one‐to‐one and confidential manner.
Peer‐led intervention	Interventions which use peer networks to ensure communication on a one‐to‐one basis, or in small and large groups
*ICT and Digital media‐based interventions*	
*Interventions which are characterized by the use of technology and digital content creation which can be disseminated over the internet or mobile networks*.	
Social media	Interventions which use social media platforms such as Facebook, YouTube, TikTok, and Instagram to disseminate information and encourage users to interact with each other and engage in dialogue on a large scale.
Mobile‐based services	Interventions which use mobile‐based services such as SMS and IVRS to disseminate information and encourage users to interact with each other and engage in dialogue on a large scale.
Digital games and learning tools	Interventions which use highly interactive and culturally relevant games and other tools through elements of roleplay and simulation to motivate users through sustained exposure.
Interactive app‐based services	Interventions which use interactive app‐based services, at scale, to disseminate information about HIV prevention and treatment and provide other resources for support.

In case the interventions studied do not exactly match the above‐mentioned categories, the team will discuss and categorize them to their closest match based on the intervention definition and make a note of the same. Additionally, if a single study covers multiple interventions, the eligible interventions will be coded and included.

#### 3.3.2 Outcome categories

The outcomes categories include behavioural and health related outcomes outlined, along the causal chain based on the above‐mentioned conceptual framework. The behavioural outcomes have been categorized at three levels – Individual (AGYW), Influencer (partner, household and community) and Institutional actors like healthcare providers. Further, the health outcomes have been divided into prevention related outcomes and research related outcomes. Subcategorization of the outcomes of the outcome have been further done based on the COM‐B model (McDonagh et al., 2018), TDF framework and the Reproductive Empowerment Framework (2017).

Table 2 summarizes the outcomes in detail.

**Table 2:** Outcome categories

Behavioural outcomes	Outcome definition
*Individual*	
Knowledge, attitudes and skills	
1. Knowledge and awareness about HIV and STI	Level of understanding around HIV transmission, diagnostics, prevention, treatment, interventions (where to seek care and how to seek care, access points, etc.)
2. HIV/STI risk perception	Beliefs that individuals have about the characteristics and risks pertaining to HIV/STIs, and intention to adopt healthy behaviour.
3. Trust in Healthcare providers/services	Level of confidence regarding available interventions and healthcare services, as well as the patient‐provider relationship (including counsellors and peer educators).
4. Individual Agency and Self Efficacy	Ability to define desires, develop plans and take decisions to execute them.
5. Negotiation and life skills	Ability to exercise voice, and choice in interactions with peers, partners, family and actors outside of immediate networks like healthcare providers, community leaders, etc.
*Influencers*	
Partner and relationship dynamics	
1. Partner's HIV/STI awareness	Measures addressing partner's knowledge of transmission, diagnostics, prevention, treatment, interventions (where to seek care and how to seek care, etc.)
2. Power equity and role in decision making	Measures addressing unequal gender and power relations
	E.g., Freedom of accessing of choice in using contraceptives, saying no to sex, etc.
3. Sexual and intimate partner violence	Measures addressing physical, verbal, and emotional abuse and harassment.
Household dynamics	
1. Parent/in‐law communication	Measures related to the ability of AGYW to have open conversations among family members.
2. Joint decision making in households	This domain has been explored through the following indicators:
	Measures specifically related to parental figures only, e.g. domestic violence, knowledge about sexual relations, intimate partner violence, use of prevention options, access to care, family planning, etc. related outcomes.
	Measures related to capacity of AGYW to be active contributors to decision making processes within the household.
	Measures related to capacity of parental figures towards creating an enabling environment for AGYW to be active contributors to decision making processes within the household.
Social and community norms	
*Social processes that help individuals change their thoughts, feelings, or behaviours*	
1. Gender norms and expectations	Measures related to the understanding and deconstruction of gender roles in the context of sexual and reproductive health, especially with regards expectations in the form of social pressure, group norms, social support, conformity, etc.
2. HIV/STI myths and misperceptions	Measures that help address or reduce HIV/STI related myths and misperceptions.
3. Stigma and discrimination	Measures that address negative opinions and behaviours towards AGYW living with, or affected by, HIV. Also include measures addressing self‐stigma.
4. Community support systems—peers, community leaders, community elders, etc.	Measures that help create an enabling ecosystem within communities through support networks. These may include CBOs, FBOs, peers, community elders, youth groups, etc.
*Institutional*	
Health care services	
*Outcomes addressing AGYW health related problems through a mix of public, private, community and voluntary health provider services*.	
1. Provider senitization and engagement skills	Measures that focus on enhancing provider knowledge and skills to make them sensitive and responsive towards the healthcare needs of AGYW.
2. Quality of care and satisfaction with services	Measures that help deliver desired health outcomes for AGYW and build user friendly services. It is also related to the continuum of care in healthcare services
*Prevention*	
Health outcomes	
1. Limiting sexual partners	Measures which promote risk reduction strategies to convey that having multiple partners is risky.
2. Correct and consistent condom use	Measures which address the correct and consistent use of condoms every time individuals engage in sexual activity with their partners.
3. Routine testing and status awareness	Measures that relate to regular HIV testing, especially for those at high risk, for them to be made aware of their HIV status and subsequently get treatment.
4. Uptake of PrEP/other Biomedical prevention options	Measures which address the uptake of PrEP (pre‐exposure prophylaxis), PEP (postexposure prophylaxis), and long acting antiretroviral (ARV) drugs and other available or upcoming prevention options.
5. Raised age of sexual debut	Measures which promote delaying sexual debut, especially for unmarried adolescent girls and women.
*Research engagement*	
1. Research awareness and benefit perception	This domain has been explored through the following indicators:
	Measures that address AGYW knowledge/awareness of HIV science, vaccines and research pipeline for new prevention options, trials and biomedical research for HIV related biomedical products.
	Measures that address influencers knowledge/awareness of HIV science, vaccines and research pipeline for new prevention options, trials of biomedical research for HIV related biomedical products.
2. Participation in biomedical research	This domain has been explored through the following indicators:
	Measures in perception change on benefits of biomedical research/clinical trials.
	Measures of AGYW's attitudes (negative/positive), beliefs and perception towards enrolment/participation in biomedical research.
	Measures of influencers attitudes (negative/positive), beliefs and perception towards enrolment/participation in biomedical research.

## 4 METHODS

### 4.1 Study design

We will include studies that assessed the effects of interventions using experimental designs or quasi‐experimental designs with nonrandom assignment that allow for causal inference. Systematic reviews on the effect of interventions will also be included.

Specifically, we will include the following:

- Studies where participants are randomly assigned to treatment and comparison group (experimental study designs).
- Studies where assignment to treatment and comparison group is based on other known allocation rules, including a threshold on a continuous variable (regression discontinuity designs) or exogenous geographical variation in the treatment allocation (natural experiments).
- Studies with nonrandom assignment to treatment and comparison group that include pre‐and posttest measures of the outcome variables of interest to ensure equity between groups on the baseline measure, and that use appropriate methods to control for selection bias and confounding. Such methods include statistical matching (e.g., propensity score matching, or covariate matching), regression adjustment (e.g., difference‐in‐differences, fixed effects regression, single difference regression analysis, instrumental variables, and ‘Heckman' selection models).
- Studies with nonrandom assignment to treatment and comparison group that include posttest measures of the outcome variables of interest only and use appropriate methods to control for selection bias and confounding, as above. This includes pipeline and cohort studies.

Ferraro and Miranda (2017) argue that combining panel data with baseline observations and statistical matching is the most effective quasi‐experimental method at reducing bias when evaluating conservation sector programmes. However, given the expected small size of the evidence base, we will include studies with post‐intervention outcome data only if they use some method to control for selection bias and confounding. To account for the differences in the quality of study designs and analysis methods, we will appraise the risk of bias in all included studies and do sub‐group analysis by risk of bias status.

Studies with both negative and positive outcomes will be considered for the above‐mentioned designs.

Before‐after studies and observational studies without control for selection bias and confounding will be excluded. Additionally, modelling‐based studies, commentaries and literature reviews will be excluded.

### 4.2 Inclusion and exclusion criteria

Studies published 2000 onwards in English language only would be considered for the EGM.

Table 3 gives an overview of the inclusion and exclusion criteria.

**Table 3:** Inclusion and exclusion criteria

Population	Adolescent girls and young women (15–24) and Influencers like parents, partners, in‐laws, healthcare providers	Sub‐Population
		Rural and Urban
		Formal and Informal
Geography	LMIC	
Topics of interest	HIV/AIDS and other STIs	
	Testing and uptake of prevention options including Condom, PrEP.	
	Consistent uptake of ART	
	Participation in Biomedical Research	
	Intimate partner violence, sexual violence or gender‐based violence affecting HIV/SRH related health outcomes	
	Behavioural factors such as relationship goals, sexual negotiations, gaps in knowledge, self‐efficacy, risk perception	
	Stigma and discrimination related barriers	
	Collective action, community support, community‐driven action	
	Social and Gender Norms affecting SRH and HIV related behaviours	
Topics not of interest	Other structural factors affecting HIV acquisition in AGYW population ‐ School attendance, labour migration, child sexual abuse, lack of economic empowerment, structural barriers to accessing sexual and reproductive health services, marriage patterns, etc.	
	Biological factors	
	Other adolescent health issues like mental health, smoking, alcoholism, drug addiction, nutrition, etc.	
	Maternal health and childcare	
	Pregnancy, Family planning (if they do not measure effects of HIV/SRH outcomes.)	
Study type	Impact Evaluation‐ RCT, Controlled Before and After, Cross‐Sectional or Panel studies, Mixed Method studies that combine any of the above design with qualitative research	
Time frame	Studies published from 2000 onwards	
Language	English	

### 4.3 Search strategy

A pilot search strategy would be developed based on the selection of studies that meet the inclusion criteria. This would be done under the guidance of an information scientists from Campbell. The search will be conducted using various databases like SCOPUS, PubMed, PsychINFO, EBSCOhost, Popline, BCI, and Compass, and review SBCC and HIV related resources available on Health Communication Capacity Collaborative (HC3). The 3ie Adolescent Sexual and Reproductive Health Evidence and Gap Map will also be searched. Moreover, we will be looking at systematic reviews and other primary studies, as well as checking the reference list of systematic reviews to identify additional studies for the EGM (Table 4).

**Table 4:** Search strategy

Population	Key terms
Adolescent girls	Girls, children, child, female child, teenagers, teens, teenaged, juveniles, youth, minor, adolescents, schoolgirl, immature, girl, adolescent, adolescence,
Young women	Women, woman, adult, female, female adult, young adult, adulthood
Sex workers	Sex work, Female sex worker, brothel, home‐based, hidden population, lodge based, street based, escort, private or phone based, window or doorway, Pimps, mediators, gharwali, madam
PLHIV	Human immunodeficiency virus, HIV positive, HIV infection, social stigma, AIDS, Acquired Immunodeficiency Syndrome, AIDS, discrimination, person living with HIV, HIV positive person, individual living with HIV, HIV prevalence, HIV screening, antiretroviral therapy, ART, tested for HIV, tested for AIDS, HIV test, immunodeficiency, immunocompromised, HIV transmission, viral load
Pregnant Women and New Mothers	HIV infection, HIV positive, pregnancy, childbirth, delivery, labour, breastfeeding, HIV treatment, HIV prevention, prevention, viral load, mother to child transmission

Settings	Key terms
Urban	Town, city, metropolitan, non‐rural, civic, municipal, urbanized
Rural	Agrarian, countryside, agricultural, local
Formal	Planning, spatial planning, urbanization, urban development, hospital, health centres (PHCs), schools, educational centre, households, self‐help groups, office spaces, religious places
Informal	Urbanization, clusters, refugee colonies, tribal areas, disaster affected areas, slums, informal housing, poverty, poor, informal settlement dweller
Lower and Middle‐Income Countries	Developing, LMIC, L&MIC, LAMI countries, LDC, transitional country, underdeveloped, low income, middle income, underserved, deprived, poor

**Influencers**	**Key terms**
Young men	children, child, teenager, teen, teenaged, juveniles, youth, young, minor, adolescent, schoolboy, male, men, young adult, adulthood, adolescent,
Family	Parents, kin, relatives, family unit, parenting, parent‐child relations, in‐laws, mother, father, family relations, intergenerational relations, immediate relations, social support, interpersonal relations, companion, married person, home, house, household, domestic, patrimonial, traditional, familial, maternal figure, paternal figure, extended family, nuclear family, blood relation, family member, blood relative
Partner	spouse, husband, sexual partners, boyfriend, sugar daddy, lover, client
Educator	Teacher, instructor, education, schoolteacher, teach, instructor, guru, principal, mentor, train, trainer, teaching staff, teaching personnel, tutor, schoolmaster, faculty, educational, educationist, guide, nurturer, advisor, educational system, education system, counsellor, professor, counselling, school
Religious leader	Religious personnel, religious leadership, spiritual leader, religious figure, priest, leader, teacher, scholar, religious person
Peers	Contemporary, friend, fellow, equal, pals,
Community elders	Community seniors, patriarchs, matriarchs
Healthcare provider	Community Health Worker (CHW), health worker, health aide, community health aide, community health promoter, community health representative, health assistant, health outreach workers, doctor, nurse, Primary Care Provider (PCP), health professional, healthcare professional, caregiver, physician, medical practitioner, heal, health, healthcare

**Intervention strategies**	**Key terms**
*Mass media*	
Print media	Press, news media, journalism, medium, print medium, publication, newspaper, paper, books, journals, comics, comic books, novel, storybook, magazines, periodicals, mass media, blog, video, news programs
Electronic media	Broadcasting, information media, media, TV, television, radio, press, mass media, FM radio, TV serials,
*Community‐Based Interventions*	
Community based, community owned, participatory, community led, community supported	
Community Media	Community radio, Local television, community video, community newspaper, newsletter, participatory radio, community screening, community supported media, local radio, digital storytelling, community photography, community storytelling
Folk Media	folk media, folklore, folktales, traditional media, lore, legend, fable, myth, culture, tale, oral tradition, oral history, storytelling, oral stories,
Theatre and arts‐based approaches	Street theatre, performance, performance art, dramatic art, stage, acting, performing, dramatics, show, entertainment, tradition, culture, storytelling, performative arts, dance theatre, participatory theatre, theatre of the oppressed, dance based, music based, drama
Capacity Strengthening	Capacity building, capacity development, empowerment, skill building, skill development, building capacity.
Community dialogues	Conversations, dialogue, discussion, conference, communication, comment, question, speeches, social
Gamification and experiential learning	Learning, immersive learning, simulated learning, observation, experimental, art, painting, performance art, fine art, arts and crafts, participatory theatre, experiential learning, protests, rally, assembly, clowning, miming, arts based public health engagement
*Interpersonal communication*	
Interindividual, inter‐social, person‐to‐person, individual, mutual	
Counselling	Therapy, guidance, advice, help, teaching, instruction, direct, recommend, advocate, counsellor, psychologist
Home Visits	Door‐to‐door, survey, house‐to‐house, canvass, direct, study, observe
Peer‐led interventions	peer conversations, discussions, dialogue, debates, meetings, interviews, peer support groups,
*ICT and digital media‐based interventions*	
Electronic media, online media, digital information, data, database, media asset, electronic medium, computerized, online version, electronic mass media, digital multimedia, electronic press	
Social Media	Social network, Facebook, Instagram, Twitter, Snapchat, WhatsApp, YouTube, TikTok, chat rooms, message boards, communication, message, Pinterest, Google Plus, WeChat, Telegram, web‐based, apps, applications, download services, blogs, forums, photo sharing, video sharing, platform, Tinder, Bumble, OkCupid, Zoom, Google Meet, etc.
Mobile Based Services	Information technology, web, voice mail, SMS, MMS, text, message, text message, IVRS, internet, e‐mail, online mail, electronic mail, message, communication, platform
Digital Games and Learning Tools	Games, electronic games, in‐app games, in‐app advertisements, virtual learning, e‐learning, program, computer program, software
Interactive App Based Services	Hands‐on, interactive, responsive, reciprocal, online, application, computer program

Outcome category	Key terms
*Knowledge, attitude and skills*	
HIV/STI knowledge and awareness	Awareness, education, health knowledge, health understanding, learning, cognition, science, familiarity, grasp, expertise, know‐how, perception, understanding, recognition, literacy, schooling, experience, insight, proficiency, comprehension, realization, attention, observation, HIV status, HIV testing, infection, risk of infection, mother‐to‐child transmission, sexual transmission, drug use, drug abuse, person living with HIV (PLHIV), screening, diagnosis, treatment, ART, medicine, Condom, lubricant, PrEP, PEP
HIV/STI risk perception	Perception, understanding, judgement, sensitivity, consciousness, insight, conception, notion, thought, recognition, concept, viewpoint, discrimination, stigma, social perception, social stigma, risk, risk‐taking, risk behaviours, risk‐reduction behaviours, attitude, idea, apprehension
Trust in healthcare services/providers	Confidence in healthcare services, patient‐provider relationships
Individual agency and self‐efficacy	Confidence, self‐confidence, faith, reliance, belief, care, dependence, obligation, responsibility, presumption, expectation, self‐assurance, self‐reliance, self‐belief, self‐motivation, accountability, liability, duty, commitment, requirement
Negotiation and life skills	Discussion, discourse, dialogue, debate, agreement, mediation, brokering, consultation, settlement, intervention, meeting, bargain, understanding, conciliation, daily life, day‐to‐day life, practical knowledge, practical skills, practical competence, life experience, elementary knowledge
*Partner‐relationship dynamics*	
Response, understanding, partnership, bond, kinship, love, involvement, interdependence, dependence, communication, interrelationship, marriage, relation, spouse, relationship, relationship‐building, family dynamics, household dynamics	
Partners HIV/STI awareness	Awareness, education, health knowledge, health understanding, learning, cognition, science, familiarity, grasp, expertise, know‐how, perception, understanding, recognition, literacy, schooling, experience, insight, proficiency, comprehension, realization, attention, observation, testing, HIV testing, HIV status, infection, risk of infection, contraception, condom, mother‐to‐child transmission, prevention, messaging, sexual values, sexual transmission, unsafe sexual practices, drug use, drug abuse, person living with HIV (PLHIV), screening, diagnosis, treatment, ART, medicine
Power equity and role in decision making	Equality, fairness, gender equity, gendered roles, household decision making, decision maker, head of household, breadwinner, head, authority, patriarchal, patriarch, integrity, justness, right, honesty, investment, fair‐mindedness, honour, share, stake, stakeholder, involvement, involve, participation, participate, active participation, passive participation, mobility, relationship
Sexual and intimate partner violence	Domestic violence, domestic abuse, domestic assault, abuse, physical abuse, mental abuse verbal abuse, verbal assault, conjugal violence, family violence, violence, force, spousal violence, spousal abuse, intra‐family violence, rape, molestation, marital rape, sexual assault, sexual harassment, sexual misconduct, sexual coercion, coercion, intimidation, aggressive behavior, relationship abuse, manipulation, controlling, violence against women, violence against children, child sexual abuse, sex crime, gender‐specific violence
*Household dynamics*	
Family, relationships, intra‐household, parents, in‐laws, mother‐in‐law, father‐in‐law, sister‐in‐law, brother‐in‐law, cousins, extended family, parentage, siblings, sister, brother, mother, father, marriage, partner, husband, spouse, loved ones, children, child, kin, relatives, relations, grandmother, grandfather, grandparents, aunt, uncle, next of kin, home, family unit, joint family, nuclear family, tradition, traditional, customs, values, contact, communication, interaction, interrelation, interrelational, cooperation, reciprocal, interdependent, dependent, dependents, head of household, patriarchal, patriarch, head of family, household head, decision maker, breadwinner, earner	
Parent/in‐law communication	Mother, father, mother‐in‐law, father‐in‐law, parents‐in‐law, kin, family, conversation, advice, guidance, help, judgement, discussion, recommendation, suggestion, disclose, disclosing, protective, protection, support, fears, facilitators, barriers, communication, sexual communication, stigma, discrimination
Joint decision making in households	Joint family, nuclear family, family, head of household, household head, breadwinner, earner, finances, financial stability, children, child, schooling, education, literacy, resolve, act, judgment making, choice making, choices, traditions, customs
*Social and community norms*	
Gender norms and expectations	Gender, gendered, masculinity, femininity, status‐quo, stigma, discrimination, gender roles, role distribution, stereotypes, stereotypical gender roles, gender stereotypes, tradition, values, customs, cultural norms, sexual roles, sexism, division of labour, delineation of roles, misogyny, gender‐specific, behavioural norms, division of responsibilities, assigning roles, allocation of duties,
HIV/STI myths and misperceptions	Misconceptions, misrepresentations, false notions, stereotypes, stereotyping preconceptions, popular beliefs, public perceptions, public opinion, prejudices, fiction, fabrication, assumptions, traditional thinking, transmission, spread, propagation
Stigma and discrimination	Prejudice, prejudicial, judgment, perception, misconception, bigotry, inequality, inequity, gender norms, sexism, discriminatory practices, narrow‐mindedness, unequal treatment, mistreatment, disparate practices
Community support systems	Aid systems, welfare, welfare schemes, community services, self‐help groups, therapy, counselling, safety net, friends, peers, family, extended family, support group, support network, network, assistance systems, helpers, aides
*Healthcare services*	
Health service, frontline health workers, frontline workers, health workers, healthcare aides, medical facilities, health facility, psychiatric facilities, health‐care, healthcare provisions, medical services, healthcare providers, healthcare professionals, doctors, nurses, psychiatrists, counsellors, therapists, healthcare organizations, assistance providers, medical providers, healthcare workforce, health staff, staff, practitioners, scientists, researchers, health personnel, caregivers, hospitals, health utilities, relief workers, healthcare centres, clinic, dispensary, healthcare institutions, medical facility, facility, government hospital, private hospital, treatment facility, public sector, private sector	
Provider sensitization and engagement skills	Raising awareness, awareness‐raising, sensitize, make aware, outreach, outreach efforts, advocate, advocacy, educate, teach, awareness‐building, informing, promote, communication, provide knowledge, informational, enhancing awareness, understanding, better understanding, recognition, involvement, listening, empathy, trust, interpersonal skills, peer‐peer communication, effective communication
Quality of care and satisfaction with services	Quality of service, quality of healthcare, quality of health services, quality of aid, quality of services provided, degree of care, standard of care, quality of provision, level of service, gratification, happiness, appreciation, personal satisfaction, patient satisfaction
*Prevention*	
Preventive, preventive measures, prophylaxis, deterrence, prevention measures, control	
Limiting sexual partners	Monogamy, intimate partner relations, safe sex, contraceptive use, abstinence, restraint, high‐risk behavior, sexual behavior
Consistent condom use	Contraception, birth control, condoms, contraception method, pregnancy prevention methods, family planning, contraceptive precautions, prophylaxis, IUD, birth control pills, abstinence, safe sex
Routine testing and status awareness	HIV testing, examinations, check‐ups, testing frequency, preventive healthcare, awareness, education, sensitizing, increased awareness, better understanding
Uptake of PrEP/other biomedical prevention options	Injection drug use, drug abuse, drug addiction, unsafe sex, preventive measures, high‐risk sexual behavior, HIV, human immunodeficiency virus, prescription, health, safety, quality of life
Raised age of sexual debut	First sexual experience, age of consent, first sexual activity, abstinence
*Research engagement*	
Research awareness and benefit perception	Understanding, knowledge, education, inquiry, scientific inquiry, self‐awareness, self‐perception, perceptions, preconceived notions, involvement, engagement, values, interest, aid
Participation in biomedical research	Community engagement, dialogues, discussions, awareness, knowledge building, community consultation, involvement, study, support, conversations, forums, meetings, learning, experiential learning, practical learning, practical training, practice, on‐site training, in‐field training, experience‐based learning, community participation, community involvement, participatory practices, community contribution, participatory

## 5 ANALYSIS AND PRESENTATION

### 5.1 Report structure

The report will include the following sections: executive summary, background, methods, results, and conclusion.

Executive summary will be a comprehensive summary of the report highlighting the key findings and future implications. The background will provide a description of the challenges faced by AGYW populations in LMICs in understanding, accessing, and negotiating information about HIV and related biomedical treatments and services. This section will also outline the objectives of the EGM and describe its scope by defining the intervention and outcomes framework, and the theory of change.

Methods would outline the data sources, inclusion and exclusion criteria and data extraction methods in detail. It would also highlight the search strategy and the PRISMA. Further, the full search strategy, including details on restrictions and filters, will be provided.

The section detailing the results will include the number, type and quality of studies retrieved for the main intervention categories, namely—mass media‐based interventions, mid media interventions, community‐based interventions, community mobilization, interpersonal/peer‐peer communication, and ICT and digital media‐based interventions. It will also include existing gaps in the framework, as well as any limitations.

The conclusion will highlight implications for researchers, policymakers and community engagement strategists on the evidence base, and the key areas to be noted for future research.

### 5.2 Filters for presentation

The results of the EGM will be presented as a matrix of interventions (*y* axis) and outcomes (*x* axis). They assess the availability of evidence across additional filters, such as country of study (using the World Bank country classifications by income), study design (e.g., RCT or systematic review), age group (15–24 years), and setting (urban formal, urban informal, rural formal and rural informal).

### 5.3 Dependency

The EGM will treat multiple reports about a single study as one study.

## 6 DATA COLLECTION AND ANALYSIS

### 6.1 Screening and study selection

Titles and abstracts of all retrieved articles will be screened by two reviewers based on intervention, study design, and population. Outcome will not be included in this process. Subsequently, full texts of eligible studies will be retrieved and screened, and the reviewers will compare the results. Should there be any conflicts, they will be resolved through discussion or by an additional reviewer. The authors of the studies will not be contacted at any point for missing information.

### 6.2 Data extraction and management

Two reviewers will extract data on both published and ongoing randomized trials related to the population, interventions, comparisons and outcomes. Coding categories (Supporting Information Appendix C) will be based on the intervention and outcome framework. Information on additional filters, like country of study (using the World Bank country classifications by income), study design (e.g., RCT or systematic review), age group (15–24 years), and setting (urban and rural and formal and informal settings, as defined by the United Nations), will be included.

### 6.3 Tools for assessing risk of bias/study quality of included reviews

As the purpose of the EGM is to identify all available Impact Evaluations, these will not be assessed (Snilstveit et al., 2017).

### 6.4 Methods for mapping

The EGM will be generated using the EPPI‐Reviewer 4 software (Eppi‐Centre, 2019).

## Supporting information

Supporting information.
