# Supplementary material for: PROTOCOL: Social and behaviour change communication interventions for strengthening HIV prevention and research among adolescent girls and young women in low‐ and middle‐income countries: An evidence and gap map
Source: Campbell Syst Rev. 2022 Jan 18;18(1):e1211. doi: 10.1002/cl2.1211 (PMC8765670; doi:10.1002/cl2.1211)
Supplement: Supplementary file 1 — Supporting information. [file CL2-18-e1211-s001.docx]

Appendices

Appendix A: Advisory Group

| **Name** | **Area of Expertise** | **Organization** |
| --- | --- | --- |
| Dr Anant Bhan | Global Health, Bioethics | Independent, Ex-President, International Association of Bioethics |
| Dr Venkatesh Chakrapani | HIV/AIDS and sexual health issues of sexual minorities | Founder and Director, C-SHaRP |
| Ms. Shruta Rawat | Gender and sexual minorities. | Research Manager, Humsafar Trust |
| Dr. Seema Sahay | HIV/AIDS Anthropology and Good Participatory Practices in Research | Scientist G, ICMR National AIDS Research Institute (NARI), Government of India |
| Dr. Aparna Khanna | Development Communication, ICT and Game design. | Associate Professor, Department of Development Communication and Extension, Lady Irwin College, Delhi University |
| Dr. Manjulika Vaz | Health Humanities, Participatory Research | Lecturer, St. John’s Research Institute |
| Dr. Tapati Dutta | Health education and Health Behaviour | Assistant Professor, Fort Lewis College |
| Manju Chatani Gada | AGYW, SRHR, Gender | AVAC |
| Hemlata Verma | Gender mainstreaming, Gender Based violence (GBV) | International Centre for Research on Women (ICRW) |
| Renu Golwalkar | Gender Equity, youth, social inclusion | Engender Health |

Appendix B: Screening Tool

Is the paper published in or beyond 2000?

Is the paper in English?

Is the paper about an intervention intended to modify the behaviour or attitudes, either directly or indirectly, of adolescent girls and young women (age 15-24) or their parents, partners, peers, community influencers like religious leaders, community elders’, professionals with whom they interact?

Is the paper based in a low- and middle-income country/countries?

Is the paper a quantitative evaluation reporting measures of eligible outcomes compared to the outcomes (1) in a comparison group (either with or without baseline outcome measures), (2) before versus after with no comparison group, or (3) a systematic review** of such studies?

Do the outcomes include measures of attitudes, beliefs or behaviour of AGYW, their influencers and partners or professionals?

Appendix C: Coding Tool

● Region

○ East Asia and Pacific

○ Europe and Central Asia

○ Latin America and Carribean

○ Middle East and North Africa

○ South Asia

○ Sub-Saharan Africa

○ Not Reported

● Country

○ Afghanistan

○ Angola

○ Armenia

○ Argentina

○ Bahrain

○ Bangladesh

○ Barbados

○ Belarus

○ Belize

○ Bolivia

○ Brazil

○ Botswana

○ Bulgaria

○ Burkina Faso

○ Cambodia

○ Cameroon

○ Chile

○ China

If just Hong Kong, use Hong King code only, NOT China

○ Congo

○ Croatia

○ Cuba

○ Colombia

○ Cyprus

○ Dominican Republic

○ Egypt

○ Ecuador

○ Eritrea

○ Estonia

○ Finland

○ Ethiopia

○ Gambia, The

○ Georgia

○ Ghana

○ Greece

○ Guatemala

○ Guinea-Bissau

○ Haiti

○ Honduras

○ Hong Kong

○ India

○ Indonesia

○ Iran

○ Ireland

○ Israel

○ Italy

○ Ivory Coast

○ Kazakhstan

○ Jordan

○ Kenya

○ Korea

○ Kuwait

○ Japan

○ Jamaica

○ Lao

○ Latin America (where multiple countries)

○ Lebanon

○ Lesotho

○ Liberia

○ Latvia

○ Lithuania

○ Luxembourg

○ Malawi

○ Madagascar

○ Malaysia

○ Mali

○ Mexico

○ Micronesia

○ Marshall Islands

○ Mozambique

○ Mongolia

○ Myanmar (Burma)

○ Namibia

○ Nepal

○ New Zealand

○ The Netherlands

○ Nicaragua

○ Niger

○ Nigeria

○ Northern Ireland

○ Pakistan

○ Norway

○ Panama

○ Papua New Guinea

○ Peru

○ Philippines

○ Poland

○ Portugal

○ Puerto Rico

○ Romania

○ Russia

○ Rwanda

○ Samoa

○ San Marino

○ Saudi Arabia

○ Scotland

○ Serbia

○ Senegal

○ Singapore

○ Sierra Leone

○ Slovakia

○ Slovenia

○ South Africa

○ Spain

○ Sri Lanka

○ Sudan

○ South Sudan

○ St Lucia

○ Swaziland

○ Syria

○ Taiwan

○ Thailand

○ Tanzania

○ Tunisia

○ Uganda

○ Ukraine

○ Turkey

○ Uzbekistan

○ Vanuatu

○ Venezuela

○ Vietnam

○ West Indies

○ Yemen

○ Zambia

○ Zimbabwe

○ St. Vincent and the Grenadines

○ Benin

○ Gabon

○ Suriname

○ El Salvador

○ Gaza

○ Not reported

● Setting

○ Rural

○ Urban

○ Formal

○ Informal

● Population

○ Adolescent girls

○ Young women

○ Sex workers

○ PLHIV

○ Pregnant women and new mothers

○ Others

● Age group

○ 15-19

○ 20-24

○ Not reported

● Influencers

○ Young men

○ Family

○ Partner

○ Educator

○ Religious Leader

○ Peers

○ Community Elders

○ Health care provider

○ Not reported

○ Others

● Study Design

○ Randomized Controlled Trial

○ Quasi-Experimental

○ Cross-Sectional or Panel Studies

with an intervention and comparison group using method to control for selection bias and confounding

○ Systematic Review

○ Process Evaluation

● Publication Status

○ Published

○ Ongoing

● Intervention strategies

○ Mass Media Interventions

■ Print Media

■ Electronic Media

○ Community-based interventions

community based, community owned, participatory, community led, community supported

■ Community Media

Community radio, Local television, community video, community newspaper, newsletter, participatory radio, community screening, community supported media, local radio, digital storytelling, community photography, community storytelling

■ Folk Media

folk media, folklore, folktales, traditional media, lore, legend, fable, myth, culture, tale, oral tradition, oral history, storytelling, oral stories,

■ Theatre and arts based approaches

Street theatre, performance, performance art, dramatic art, stage, acting, performing, dramatics, show, entertainment, tradition, culture, story-telling, performative arts, dance theatre, participatory theatre, theatre of the oppressed, dance based, music based, drama

■ Community Dialogues

■ Capacity Strengthening

■ Gamification and Experiential Learning

Learning, immersive learning, simulated learning, observation, experimental, art, painting, performance art, fine art, arts and crafts, participatory theatre, experiential learning, protests, rally, assembly, clowning, miming, arts based public health engagement

○ Interpersonal Communication

Interindividual, inter-social, person-to-person, individual, mutual, communicative

■ Counselling (One-on-One/ Couple Counselling)

■ Home Visits

Door-to-door, survey, house-to-house, canvass, direct, study, observe

■ Peer-led intervention

peer conversations, discussions, dialogue, debates, meetings, interviews, peer support groups,

○ ICT and Digital Media based interventions

Electronic media, online media, digital information, data, database, media asset, electronic medium, computerized, online version, electronic mass media, digital multimedia, electronic press

■ Social Media

Social network, Facebook, Instagram, Twitter, Snapchat, Whatsapp, Youtube, TikTok, chat rooms, message boards, communication, message, Pinterest, Google Plus, WeChat, Telegram, web-based, apps, applications, download services, blogs, forums, photo sharing, video sharing, platform

■ Mobile Based Services

Information technology, web, voice mail, SMS, MMS, text, message, text message, IVRS, internet, e-mail, online mail, electronic mail, message, communication, platform

■ Digital Games and Learning Tools

Games, electronic games, in-app games, in-app advertisements, virtual learning, e-learning, program, computer program, software

■ Interactive App Based Services

Hands-on, interactive, responsive, reciprocal, online, application, computer program

● Outcomes

○ Knowledge, attitude and skills

■ Knowledge and awareness about HIV/STI

Awareness, education, health knowledge, health understanding, learning, cognition, science, familiarity, grasp, expertise, know-how, perception, understanding, recognition, literacy, schooling, experience, insight, proficiency, comprehension, realization, attention, observation, HIV status, HIV testing, infection, risk of infection, mother-to-child transmission, sexual transmission, drug use, drug abuse, person living with HIV (PLHIV), screening, diagnosis, treatment, ART, medicine

■ HIV/STI risk perception

Perception, understanding, judgment, sensitivity, consciousness, insight, conception, notion, thought, recognition, concept, viewpoint, discrimination, stigma, social perception, social stigma, risk, risk-taking, risk behaviours, risk-reduction behaviours, attitude, idea, apprehension

■ Trust in healthcare providers/services

■ Individual agency and self-efficacy

Confidence, self-confidence, faith, reliance, belief, care, dependence, obligation, responsibility, presumption, expectation, self-assurance, self-reliance, self-belief, self-motivation, accountability, liability, burden, duty, commitment, requirement

■ Negotiation and life skills

Discussion, discourse, dialogue, debate, agreement, mediation, brokering, consultation, settlement, intervention, meeting, bargain, understanding, conciliation, daily life, day-to-day life, practical knowledge, practical skills, practical competence, life experience, elementary knowledge

○ Partner and relationship dynamics

Response, understanding, partnership, bond, kinship, love, involvement, interdependence, dependence, communication, interrelationship, marriage, relation, spouse, relationship, relationship-building, family dynamics, household dynamics

■ Partner's HIV/STI awareness

Awareness, education, health knowledge, health understanding, learning, cognition, science, familiarity, grasp, expertise, know-how, perception, understanding, recognition, literacy, schooling, experience, insight, proficiency, comprehension, realization, attention, observation, testing, HIV testing, HIV status, infection, risk of infection, contraception, condom, mother-to-child transmission, prevention, messaging, sexual values, sexual transmission, unsafe sexual practices, drug use, drug abuse, person living with HIV (PLHIV), screening, diagnosis, treatment, ART, medicine

■ Power equity and role in decision making

Equality, fairness, gender equity, gendered roles, household decision making, decision maker, head of household, breadwinner, head, authority, patriarchal, patriarch, integrity, justness, right, honesty, investment, fair-mindedness, honour, share, stake, stakeholder, involvement, involve, participation, participate, active participation, passive participation, mobility, relationship

■ Sexual and intimate partner violence

Domestic violence, domestic abuse, domestic assault, abuse, physical abuse, mental abuse verbal abuse, verbal assault, conjugal violence, family violence, violence, force, spousal violence, spousal abuse, intra-family violence, rape, molestation, marital rape, sexual assault, sexual harassment, sexual misconduct, sexual coercion, coercion, intimidation, aggressive behaviour, relationship abuse, manipulation, controlling, violence against women, violence against children, child sexual abuse, sex crime, gender-specific violence

○ Household dynamics

Family, relationships, intra-household, parents, in-laws, mother-in-law, father-in-law, sister-in-law, brother-in-law, cousins, extended family, parentage, siblings, sister, brother, mother, father, marriage, partner, husband, spouse, loved ones, children, child, kin, relatives, relations, grandmother, grandfather, grandparents, aunt, uncle, next of kin, home, family unit, joint family, nuclear family, tradition, traditional, customs, values, contact, communication, interaction, interrelation, interrelational, cooperation, reciprocal, interdependent, dependent, dependents, head of household, patriarchal, patriarch, head of family, household head, decision maker, breadwinner, earner

■ Parent/in-law communication

Mother, father, mother-in-law, father-in-law, parents-in-law, kin, family, conversation, advice, guidance, help, judgement, discussion, recommendation, suggestion, disclose, disclosing, protective, protection, support, fears, facilitators, barriers, communication, sexual communication, stigma, discrimination

■ Joint decision making in households

Joint family, nuclear family, family, head of household, household head, breadwinner, earner, finances, financial stability, children, child, schooling, education, literacy, resolve, take action, judgment making, choice making, choices, traditions, customs

○ Social and Community norms

■ Gender norms and expectations

Gender, gendered, masculinity, femininity, status-quo, stigma, discrimination, gender roles, role distribution, stereotypes, stereotypical gender roles, gender stereotypes, tradition, values, customs, cultural norms, sexual roles, sexism, division of labour, delineation of roles, misogyny, gender-specific, behavioural norms, division of responsibilities, assigning roles, allocation of duties,

■ HIV/STI myths and misperceptions

Misconceptions, misrepresentations, false notions, stereotypes, stereotyping preconceptions, popular beliefs, public perceptions, public opinion, prejudices, fiction, fabrication, assumptions, traditional thinking, transmission, spread, propagation

■ Stigma and discrimination

Prejudice, prejudicial, judgment, perception, misconception, bigotry, inequality, inequity, gender norms, sexism, discriminatory practices, narrow-mindedness, unequal treatment, mistreatment, disparate practices

■ Community support systems

Aid systems, welfare, welfare schemes, community services, self-help groups, therapy, counseling, safety net, friends, peers, family, extended family, support group, support network, network, assistance systems, helpers, aides

○ Healthcare services

Health service, frontline health workers, frontline workers, health workers, healthcare aides, medical facilities, health facility, psychiatric facilities, health-care, healthcare provisions, medical services, healthcare providers, healthcare professionals, doctors, nurses, psychiatrists, counsellors, therapists, healthcare organizations, assistance providers, medical providers, healthcare workforce, health staff, staff, practitioners, scientists, researchers, health personnel, caregivers, hospitals, health utilities, relief workers, healthcare centers, clinic, dispensary, healthcare institutions, medical facility, facility, government hospital, private hospital, treatment facility, public sector, private sector

■ Provider sensitization and engagement skills

Raising awareness, awareness-raising, sensitize, make aware, outreach, outreach efforts, advocate, advocacy, educate, teach, awareness-building, informing, promote, communication, provide knowledge, informational, enhancing awareness, understanding, better understanding, recognition, involvement, listening, empathy, trust, interpersonal skills, peer-peer communication, effective communication

■ Quality of care/satisfaction with services

Quality of service, quality of healthcare, quality of health services, quality of aid, quality of services provided, degree of care, standard of care, quality of provision, level of service, gratification, happiness, appreciation, personal satisfaction, patient satisfaction

○ Prevention

Preventive, preventive measures, prophylaxis, deterrence, prevention measures, control

■ Correct & Consistent condom use

Contraception, birth control, condoms, contraception method, pregnancy prevention methods, family planning, contraceptive precautions, prophylaxis, IUD, birth control pills, abstinence, safe sex

■ Routine testing and status awareness

HIV testing, examinations, check-ups, testing frequency, preventive healthcare, awareness, education, sensitizing, increased awareness, better understanding

■ Uptake of PrEP/ other biomedical prevention options

Injection drug use, drug abuse, drug addiction, unsafe sex, preventive measures, high-risk sexual behaviour, HIV, human immunodeficiency virus, prescription, health, safety, quality of life

■ Limiting sexual partners

Monogamy, intimate partner relations, safe sex, contraceptive use, abstinence, restraint, high-risk behaviour, sexual behaviour

■ Raised age of sexual debut

○ Research engagement

■ Research awareness and benefit perception

Understanding, knowledge, education, inquiry, scientific inquiry, self-awareness, self-perception, perceptions, preconceived notions, involvement, engagement, values, interest, aid

■ Participation in biomedical research

Community engagement, dialogues, discussions, awareness, knowledge building, community consultation, involvement, study, support, conversations, forums, meetings, learning, experiential learning, practical learning, practical training, practice, on-site training, in-field training, experience-based learning, community participation, community involvement, participatory practices, community contribution, participatory

● Year of Publication

○ 2000-2004

○ 2005-2009

○ 2010-2014

○ 2015-2019

○ 2020-2021

● Funding Agency

○ 3ie

○ AusAid

○ BMGF

○ Canadian International Development Agency (CIDA)

○ Canadian Institutes for Health Research

○ Center for Disease Control and Prevention

○ Center for Interdisciplinary Research on AIDS (CIRA)

○ DFID UK

○ Duke Center for AIDS Research

○ Emory Center for AIDS Research

○ European Commission

○ ESRC, UK

○ Fogarty International Centre

○ Ford Foundation

○ Global Fund to Fight AIDS, Tuberculosis and Malaria

○ German Technical Corporation of DeutscheGesellschaft fuer Technische Zusammenerbeit (GTZ)

○ IAVI

○ IDRC, Canada

○ Irish Aid

○ National Institute on Alcohol Abuse and Alcoholism (NIAAA)

○ National Institute of Allergy and Infectious Diseases

○ National Institute of Child Health and Human Development

○ National Institute on Drug Abuse (NIDA)

○ NIH

○ NIMH

○ National Institute of Nursing Research

○ President's Emergency Plan for AIDS Relief

○ Rockefeller Foundation

○ Sigrid Rausing Trust

○ South African Medical Research Council

○ Stephen Lewis Foundation

○ Swedish International Development Cooperation Agency (SIDA)

○ UK Medical Research Council

○ UNAIDS

○ UNDP

○ UNFPA

○ UNICEF

○ USAID

○ Wellcome Trust

○ WHO

○ World AIDS Foundation

○ World Bank

○ Not Reported

○ Not received any funding

● Funding Agency (other)

● AMSTAR-2

○ 1. Did the research questions and inclusion criteria for the review include the components of PICO?

■ Yes

■ No

○ 2*. Did the report of the review contain an explicit statement that the review methods were established prior to the conduct of the review and did the report justify any significant deviations from the protocol?

■ Yes

■ Partially Yes

■ No

○ 3. Did the review authors explain their selection of the study designs for inclusion in the review?

■ Yes

■ No

○ 4*. Did the review authors use a comprehensive literature search strategy?

■ Yes

■ Partially Yes

■ No

○ 5. Did the review authors perform study selection in duplicate?

■ Yes

■ No

○ 6. Did the review authors perform data extraction in duplicate?

■ Yes

■ No

○ 7*. Did the review authors provide a list of excluded studies and justify the exclusions?

■ Yes

■ Partially Yes

■ No

○ 8. Did the review authors describe the included studies in adequate detail?

■ Yes

■ Partially Yes

■ No

○ 9*. Did the review authors use a satisfactory technique for assessing the risk of bias (RoB) in individual studies that were included in the review?

■ Yes

■ Partially Yes

■ No

○ 10. Did the review authors report on the sources of funding for the studies included in the review?

■ Yes

■ No

○ 11*. If meta-analysis was performed, did the review authors use appropriate methods for statistical combination of results?

■ Yes

■ No

■ No meta-analysis conducted

○ 12. If meta-analysis was performed, did the review authors assess the potential impact of RoB in individual studies on the results of the meta-analysis or other evidence synthesis?

■ Yes

■ No

■ No meta-analysis conducted

○ 13*. Did the review authors account for RoB in primary studies when interpreting/discussing the results of the review?

■ Yes

■ No

○ 14. Did the review authors provide a satisfactory explanation for, and discussion of, any heterogeneity observed in the results of the review?

■ Yes

■ No

○ 15*. If they performed quantitative synthesis did the review authors carry out an adequate investigation of publication bias (small study bias) and discuss its likely impact on the results of the review?

■ Yes

■ No

■ No Meta-analysis conducted

○ 16. Did the review authors report any potential sources of conflict of interest, including any funding they received for conducting the review?

■ Yes

■ No

○ Overall

■ High

No or one non-critical weakness

■ Moderate

More than one non-critical weakness

■ Low

One critical flaw with or without non-critical weaknesses

■ Critically Low

More than one critical flaw with or without non-critical weaknesses

● Study confidence

○ Low confidence SRs

○ Medium confidence SRs

○ High confidence SRs

○ Impact Evaluations
